# The Lipid Line of Defence: Monounsaturated Fatty Acids (MUFAs) in Cancer and Ferroptosis

**DOI:** 10.1155/omcl/9604592

**Published:** 2026-10-13

**Authors:** Sneha D. Baadkar, Amoolya Kandettu, Swagat Ray, Sanjiban Chakrabarty

**Affiliations:** ^1^ Department of Public Health Genomics Manipal School of Life Sciences Manipal Academy of Higher Education Manipal India manipal.edu; ^2^ School of Natural Sciences College of Health and Science University of Lincoln Lincoln LN6 7TS UK lincoln.ac.uk

**Keywords:** FASN, ferroptosis, lipid metabolism, MUFA, redox homeostasis, SCD1

## Abstract

**Background:**

Monounsaturated fatty acid (MUFA) metabolism promotes cancer progression and survival by regulating membrane lipid composition, maintaining lipid homeostasis and preserving redox balance. A growing body of evidence indicates that altered MUFA metabolism promotes tumour growth while protecting cancer cells from ferroptosis.

**Aim:**

This review summarises current knowledge on the contributions of MUFA metabolism to carcinogenesis, regulation of ferroptosis and treatment response across different cancers. Further, we discussed the emerging treatment strategies targeting MUFA metabolic pathways.

**Methods:**

Relevant experimental and clinical studies investigating MUFA metabolism, lipid metabolic reprogramming, ferroptosis and therapeutic interventions in cancer were comprehensively reviewed and critically analysed.

**Results:**

Enhanced de novo MUFA synthesis and incorporation into membrane phospholipids increase the MUFA‐to‐polyunsaturated fatty acid (PUFA) ratio, thereby reducing the availability of oxidation‐prone PUFAs, limiting lipid peroxidation, and protecting cancer cells from ferroptosis. MUFA‐driven membrane remodelling enhances membrane fluidity and activates oncogenic signalling pathways, including epithelial‐mesenchymal transition (EMT), cancer cell migration, invasion and metastasis. These effects are mediated by the dysregulated expression of key lipogenic enzymes and signalling pathways that reprogramme lipid metabolism to meet the metabolic demands of cancer cells. Multiple studies have shown that MUFA metabolism is a promising therapeutic target. Furthermore, the inhibition of stearoyl‐CoA desaturase‐1 (SCD1) and fatty acid synthase (FASN), key enzymes in MUFA metabolism, sensitises cancer cells to ferroptosis by enhancing lipid peroxidation.

**Conclusion:**

MUFA metabolism is a key regulator of cancer progression and ferroptosis resistance. Targeting MUFA metabolic pathways is a promising therapeutic strategy. However, mechanistic studies are required to elucidate the molecular mechanisms underlying sensitivity to ferroptosis and to optimise clinical applications.

**Summary**

1. Monounsaturated fatty acids (MUFAs) drive cancer progression through metabolic reprogramming.
2. Oncogenic signalling rewires the MUFA metabolism in cancer cells.
3. MUFAs suppress ferroptosis by the inhibition of lipid peroxidation.
4. Stearoyl‐CoA desaturase‐1 (SCD1) and fatty acid synthase (FASN) are promising targets for cancer therapy.
5. Targeting MUFA metabolism may overcome chemoresistance.

## 1. Introduction

Lipid metabolism plays a central role in cancer biology, dictating key processes such as tumour initiation, tumour growth, metastasis and drug resistance [1, 2]. Among the diverse classes of lipids, monounsaturated fatty acids (MUFAs), characterised by a single carbon–carbon double bond (C═C) in their fatty acyl (hydrocarbon) chains, have attracted increasing attention for their multifaceted roles in tumour metabolism and survival [3]. These fatty acids, including oleic acid (OA) (C18:1) and palmitoleic acid (PA) (C16:1), are either obtained from the diet or synthesised endogenously through the action of stearoyl‐CoA desaturase‐1 (SCD1), a rate‐limiting enzyme in MUFA biosynthesis. MUFA metabolism contributes significantly to membrane lipid composition, energy storage and redox homeostasis. Moreover, the role of MUFAs has been extensively characterised in the regulation of ferroptosis, an iron‐dependent form of regulated cell death, driven by excessive lipid peroxidation. Increased incorporation of MUFAs into membrane phospholipids reduces the abundance of oxidation‐prone PUFAs, thereby limiting lipid peroxidation and suppressing ferroptosis [4, 5].

Cancer cells exhibit increased de novo lipogenesis, for example, increased MUFA production, to mitigate the augmented requirement for membrane biogenesis and metabolic adaptation during stress [6, 7]. MUFAs are preferentially incorporated into membrane phospholipids, thereby regulating membrane fluidity and stability. This process reduces lipid peroxidation, prevents oxidative damage and protects cancer cells from ferroptotic cell death [8]. Compared with polyunsaturated fatty acids (PUFAs), which are highly susceptible to lipid peroxidation and induce ferroptosis, MUFAs play a protective role by blocking the accumulation of lipid‐derived ROS, thereby ensuring cancer cell viability under oxidative stress [9]. Emerging evidence indicates that MUFA metabolism intersects with several oncogenic signalling pathways implicated in the molecular pathogenesis of cancer, including PI3K/AKT/mTOR, Wnt/β‐catenin, AMP‐activated kinase (AMPK), MYC and EGFR. These pathways activate transcriptional regulators such as sterol regulatory element‐binding proteins (SREBPs) and peroxisome proliferator‐activated receptors (PPARs), which co‐ordinately upregulate lipogenic enzymes including ATP citrate lyase (ACLY), acetyl‐CoA carboxylase (ACC), fatty acid synthase (FASN) and SCD1. The resulting increase in fatty acid desaturation and MUFA biosynthesis promotes membrane biogenesis, metabolic adaptation, redox homeostasis, ferroptosis resistance and sustained tumour growth and metastasis [10, 11].

Mechanistic evaluation of the context‐dependent effects of MUFA metabolism in cancer pathophysiology is critical to elucidate its dual role in promoting tumour development and preventing ferroptosis [4]. In this review, we aim to systematically explore the role of MUFA metabolism in cancer progression, with particular emphasis on MUFA biosynthesis, uptake and incorporation into cellular lipids, as well as their regulation by lipogenic enzymes and oncogenic signals. We also highlighted how changes in MUFA metabolism affect ferroptosis sensitivity, suggesting potential treatment approaches that target fatty acid desaturation enzymes to induce ferroptosis and overcome drug resistance. In addition, we outline the potential and the challenges of using MUFA metabolism as a therapeutic target for anticancer treatment, offering fresh perspectives on lipid‐targeted cancer treatments.

## 2. Biosynthesis of MUFAs

Although fatty acid synthesis begins in the cytosol, the endoplasmic reticulum (ER) is the principal site of fatty acid desaturation, phospholipid synthesis and membrane lipid remodelling, which are essential for maintaining cellular homeostasis [12]. However, the precise location of lipid synthesis varies according to the lipid type, followed by their modification and sorting in the Golgi apparatus [12]. Phospholipids, fatty acids, sterols, sphingolipids and acylglycerols make up the majority of lipids in mammals [13]. Endogenous lipid synthesis and dietary intake work together to maintain the lipid pool in both healthy and cancerous cells [14–16]. Predominantly, MUFAs are generally produced de novo, and PUFAs are obtained from the diet in normal cells. MUFAs are characterised by fatty acid carbon chains that consist of two carbon atoms linked by a single double bond [4, 17].

### 2.1. Exogenous MUFA Uptake

During exogenous uptake of MUFAs from the diet, these fatty acids are absorbed by intestinal enterocytes within micelles and subsequently secreted as chylomicrons into the lymphatic and blood circulation [18]. This then is captured by the liver, where they are assembled into very low‐density lipoproteins (VLDLs) and then into LDLs, where some of these lipids are distributed to other organs, while the uncaptured chylomicrons form high‐density lipoproteins (HDLs) [4, 19]. Lipoproteins are composed of cholesterol, triglycerides and proteins [20]. Triglycerides incorporated into these lipoproteins are further cleaved by the enzyme lipoprotein lipase present at the membranes of epithelial cells as free fatty acids (FFAs). FFAs produced are subsequently taken up by cells via various transporters, such as fatty acid transport proteins (FATPs), fatty acid translocase (FAT/CD36), and solute carrier protein family 27 (SLC27). The absorbed FFAs are quickly converted into fatty acyl‐CoA by ACSL family enzymes to be further incorporated into the cell membrane [4] (Figure 1).

**Figure 1:**
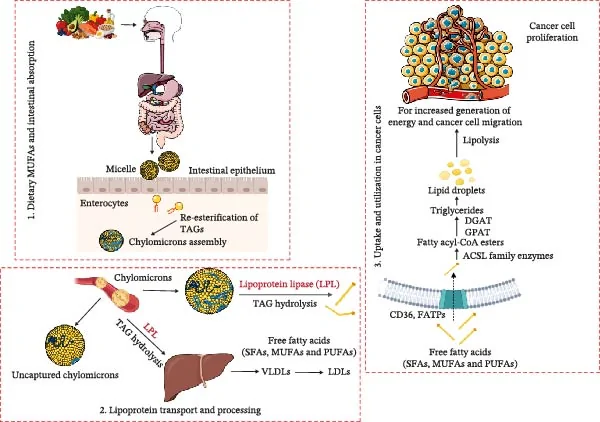
Exogenous uptake of MUFA in the body. Lipids from the diet are absorbed by intestinal cells as micelles in the body, then secreted as chylomicrons into the blood, which are further processed in the liver into HDL, LDL and VLDL. An enzyme, lipoprotein lipase, cleaves these lipoproteins into triglycerides and glycerols, which are taken up by cells as free fatty acids or further stored in the lipid droplets. In cancer cells, this breakdown is increased for active cell proliferation and migration.

### 2.2. *De Novo* MUFA Biosynthesis

The de novo synthesis of MUFAs mainly occurs in the cytoplasm of hepatocytes or adipocytes [21]. Glucose taken up by cells is metabolised to pyruvate, which enters the tricarboxylic acid (TCA) cycle in the mitochondria to generate reducing equivalents such as NADH and FADH2 [5]. Within the mitochondria, citrate is formed by the condensation of acetyl‐CoA and oxaloacetate (OAA) [22]. Citrate is then exported to the cytoplasm via the mitochondrial citrate carrier (CIC/SLC25A1), an integral protein of the inner mitochondrial membrane that facilitates the exchange of citrate with other tricarboxylates or dicarboxylates. In the cytoplasm, citrate is converted back into acetyl‐CoA by ACLY, which serves as a pivotal substrate for fatty acid synthesis [23]. ACC catalyses the ATP‐dependent carboxylation of acetyl‐CoA to form malonyl‐CoA, marking the rate‐limiting step in the fatty acid biosynthetic pathway. Acetyl‐CoA also serves as a precursor for cholesterol synthesis via the mevalonate pathway. The elongation of the carbon chain occurs via a condensation reaction between acetyl‐CoA and malonyl‐CoA, catalysed by FASN, a multifunctional enzyme complex comprising seven catalytic domains. This reaction ultimately produces saturated fatty acids (SFAs), such as palmitic acid (C16:0), utilising acetyl‐CoA as a primer, malonyl‐CoA as a two‐carbon donor and NADPH as a reducing cofactor [24, 25]. SCD, an iron‐ and oxygen‐dependent enzyme, is the primary enzyme that catalyses the synthesis of double bonds in SFA via desaturation. SCD located in the ER exhibits Δ^9^‐desaturase activity, creating a double bond in the saturated fatty acyl‐CoAs, like palmitoyl‐CoA (C16:0) and stearoyl‐CoA (C18:0), between 9 and 10 carbon atoms from the carboxylic group to form MUFAs like PA (C16:1) and OA (C18:1) [26]. Elongase enzymes belonging to the elongation of very long‐chain fatty acids (ELOVL) family facilitate the further elongation of C16 and C18 MUFAs into very‐long‐chain fatty acids, such as stearic acid (C18:0) and arachidic acid (C20:0). Among the seven identified isoforms (ELOVL1–7), ELOVL1, ELOVL3, ELOVL6 and ELOVL7 preferentially elongate SFAs and MUFAs, which are first converted into their activated forms, fatty acyl‐CoA derivatives, by long‐chain acyl‐CoA synthetase (ACSL) enzymes [27]. Further, with the action of carnitine palmitoyltransferase 1 (CPT1), fatty acyl‐CoA is converted into fatty acyl‐carnitine in the outer mitochondrial membrane (OMM), which is then transported into the mitochondrial matrix by the carnitine‐acylcarnitine translocase (CACT) working on the inner mitochondrial membrane (IMM). Within the mitochondria, these fatty acyl‐CoAs undergo β‐oxidation via the fatty acid oxidation (FAO) pathway, yielding acetyl‐CoA, which enters the TCA cycle, thereby linking lipid metabolism to energy production [28]. Most of the MUFAs are integrated into membrane phospholipids, which subsequently influence the properties of the cell membrane, and a few are further processed for the production of PUFAs, hence forming a lipid pool in the ER (Figure 2).

**Figure 2:**
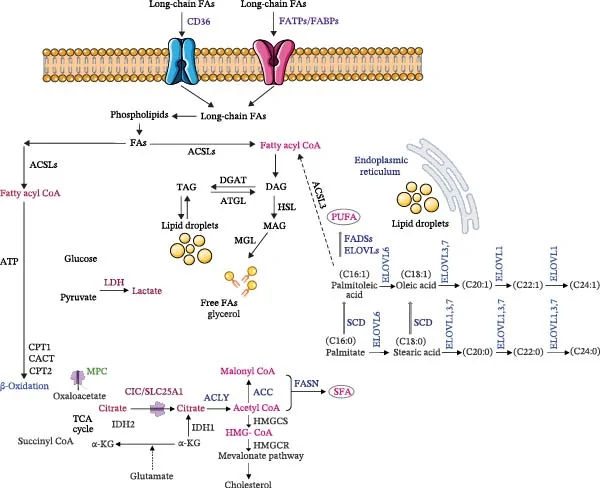
Schematic representation of the de novo biosynthesis and metabolic fate of monounsaturated fatty acids (MUFAs) in mammalian cells. The schematic illustration explaining the cellular pathways involved in MUFA synthesis and utilisation. Glucose‐derived pyruvate enters the TCA cycle in mitochondria, generating citrate, which is exported to the cytoplasm via the mitochondrial citrate carrier (CIC/SLC25a1). Cytosolic citrate is cleaved by ATP citrate lyase (ACLY) to produce acetyl‐CoA, the precursor for fatty acid synthesis. Acetyl‐CoA is converted to malonyl‐CoA by acetyl‐CoA carboxylase (ACC), and fatty acid synthase (FASN) catalyses the production of saturated fatty acids such as palmitate (C16:0). Stearoyl‐CoA desaturase (SCD) introduces a double bond, forming MUFAs like palmitoleic acid (C16:1) and oleic acid (C18:1). These MUFAs can be further elongated by ELOVL enzymes to form long‐ and very‐long‐chain fatty acids. MUFAs are incorporated into complex lipids, including phospholipids, triglycerides and lipid droplets, or are used in PUFA biosynthesis. Fatty acid uptake is mediated by CD36, FATPs and FABPs, and activated by ACSL for further metabolism.

## 3. MUFA Turnover in Cancer Cells

De novo lipogenesis is often accelerated in cancer cells, thereby increasing MUFA biosynthesis. Stearic acid (C18:0) is transformed into OA (C18:1) by SCD1, which catalyses the incorporation of a double bond into saturated fatty acyl‐CoAs. Elevated SCD1 expression has been observed in various cancers and correlates with increased MUFA levels and tumour aggressiveness [3, 4]. Studies have shown that increased SCD1 activity in KRAS‐mutant cancer models leads to a higher MUFA:SFA ratio, thereby enhancing membrane fluidity and oxidative stress tolerance [29]. In addition to producing their own MUFAs, cancer cells also trigger the uptake of exogenous MUFAs via upregulation of fatty acid transporters such as CD36, plasma membrane fatty acid‐binding proteins (FABPpms) and FATPs [30]. MUFA turnover is the hallmark of cancer cell metabolic reprogramming, enabling rapid growth, survival under metabolic stress and drug resistance. Pharmacological inhibition of MUFA biosynthesis or uptake is a highly promising therapeutic target but one that must account for tumour cell metabolic plasticity and compensatory mechanisms [4].

## 4. Role of MUFAs in Carcinogenesis

MUFAs have recently gained attention for their multifaceted roles in cancer biology, particularly in dysregulated lipid metabolism. While MUFAs were previously suspected to be merely structural components of cell membranes and energy storage molecules, several studies have also shown that increased MUFA uptake can enhance cell growth and proliferation [2, 4]. Thus, increased incorporation of MUFAs into membrane phospholipids protects cancer cells from ferroptosis by displacing oxidation‐prone PUFAs [8]. This reduces iron‐dependent lipid peroxidation, limits the accumulation of toxic lipid hydroperoxides, preserves membrane integrity and ultimately enhances cancer cell survival.

### 4.1. Cancer Risk Associated With Diet‐Derived MUFA

A high‐fat diet is recognised as an important risk factor for carcinogenesis by promoting obesity‐associated chronic inflammation, insulin resistance, oxidative stress, lipid metabolic reprogramming and increased availability of fatty acids [31]. These changes activate oncogenic signalling pathways, enhancing tumour progression, remodelling the tumour microenvironment and facilitating tumour initiation, progression and metastasis. OA (18:1,n‐9) is the most commonly found MUFA in the diet, specifically in olive oil [32]. Studies on dietary MUFA intake reported mixed findings regarding cancer risk (Table 1).

**Table 1:** Dietary MUFAs and their association with cancer risk.

MUFA source	Cancer type	Cancer risk	References
Olive oil, nuts and MUFA enriched food	Breast cancer	Increased	[33]
Olive oil	Breast cancer and cancer of digestive system	Decreased	[34]
Olive oil	All types of cancer	Neutral	[35]
Red meat and processed meat, dairy products, butter and poultry	Colorectal cancer	Increased	[36]
Total fatty acid (TFA) intake in food	Oesophageal squamous cell carcinoma	Decreased	[15]

### 4.2. Altered MUFA Metabolism Genes in Cancer Progression

Tumour cells can channel the excess glycolytic byproduct pyruvate into de novo fatty acid synthesis, ensuring a continuous supply of lipids and their precursors necessary for membrane formation in a rapidly proliferating cell population [5]. A significant portion of fatty acids synthesised endogenously in cancer cells is esterified into phospholipids and subsequently incorporated into the membranes of proliferating tumour cells [37]. This increase in lipogenesis observed in tumour cells is accompanied by the increased activity and expression levels of various lipogenic enzymes. Hence, the expression of lipogenic genes is directly correlated with cancer aggressiveness, staging and treatment resistance [25]. The genes altered in cells that increase cancer risk are found to promote cellular proliferation and cancer growth and are discussed below (Table 2).

**Table 2:** Genes and enzymes regulating MUFA metabolism in cancer.

Gene	Function	Alteration in cancer	Impact on tumour progression	References
ATP citrate lyase (ACLY)	Conversion of cytosolic citrate to acetyl‐CoA	Gene knockdown by RNAi	Growth arrest in lung cancer	[38]
		Silencing of ACLY by short hairpin RNA increased expression SREBP and alternatively ACSS2 expression producing more acetyl‐CoA	Decrease proliferation and induces apoptosis in various cancer cell lines including PC‐3M (prostate cancer), HepG2 (liver cancer), HOP62 (lung cancer) cell lines	[39]
		ACLY knockdown impaired glucose dependent lipid synthesis	Decreased cytokine‐stimulated cell proliferation in FL5.12 cells a murine pro‐B lymphocyte cell line	[40]
		Knockdown of ACLY	Decreased PM2.5‐induced EMT in in vitro and in vivo in human bronchial epithelial (HBE) cell line of lung cancer	[41]
		Acetylation at specific lysines of ACLY gene	Stabilised lipid synthesis and increased lung cancer proliferation	[42]
Acetyl‐CoA carboxylase (ACC)	Conversion of acetyl‐CoA to produce malonyl‐CoA	ACC1/ACC2 inhibition as well as dual siRNA knockdown	Decreased cell proliferation in glioblastoma	[43]
		TGF‐β‐activated kinase mediated ACC phosphorylation	Inhibits lipogenic activity and promotes EMT in breast cancer	[44]
Fatty acid synthase (FASN)	Production of saturated fatty acids (SFA) from acetyl‐coenzyme A (CoA) and malonyl‐CoA	FASN KO increased SREBP2	Increased proliferation in HCC cell lines and in vivo	[45]
		USP2a deubiquitinating enzyme stabilises FASN and Mdm2	Induces apoptotic resistance in prostate cancer	[46]
Stearoyl‐CoA desaturase (SCD)	Converts SFA to MUFAs by desaturation	SCD gene interference and SCD inhibitor A939572	Reduced cell proliferation and invasion in urinary bladder cancer	[47]
		Regulation of SCD1 by ChREBP	Showed increased SCD1 expression facilitating invasion and migration in colorectal cancer	[48]
		Silencing of SCD1 by RNAi	Reduces ERK1/2 signalling and inhibits β‐catenin target gene activation decreasing proliferation and EMT in breast cancer	[49]
Elongation of very‐long‐chain fatty acids (ELOVLs)	Elongates the carbon chains of fatty acids into very long chain fatty acids	Based on survival analysis and differential expression analysis of ELOVL1 and ELOVL3	The poor prognosis of HCC associated with liver cancer. ELOVL1 interfere with immune checkpoint inhibitors and associated with TP53 oncogenic mutation	[50]
		Based on survival analysis, differential analysis and clinical correlation analysis of ELOVL3	Overexpression of ELOVL3 showed poor overall survival of patients with HCC	[51]
		Knockdown of ELOVL6	Shows Akt activation and reduced cell proliferation in liver cancer	[52]
		Knockdown of ELOVL7	Decreased proliferation in prostate cancer and affected de novo synthesis of androgen	[53]
Long‐chain acyl‐CoA synthetase (ACSL3)	Conversion of fatty acids to fatty acyl‐CoA esters	Regulation of ACSL3 gene by OCT1	Reduced in vivo growth of prostate cancer	[54]
Sterol regulatory element binding protein 1 (SREBP1)	Transcription factor	SREBP1 regulates MUFA genes such as ACC, FASN, SCD through the NF‐κB‐MMP7 axis through increased ROS	Promotes the invasion of colon cancer cells	[10]
Carbohydrate response element binding protein (ChREBP)	Transcription factor	siRNA‐mediated ChREBP knockdown, ChREBP regulating the expression of ELOVL6 and SCD1	Inhibition of cell proliferation and induction of apoptosis in colon cancer	[55]
Peroxisome proliferator‐activated receptor α (PPARα) Hypoxia‐inducible factor‐2 α (HIF‐2 α)	Transcription co‐factor	On PPARδ activation binds to MUFA genes and NANOG promoter	Promotes development of colorectal cancer metastasis	[56]
	Transcription factor	Knockdown of either HIF‐2 α or SCD1	Shows apoptosis and reduced colony formation ability and cell migration in clear cell renal cell carcinoma (ccRCC)	[57]

#### 4.2.1. ACLY

ACLY is a key metabolic enzyme that connects carbohydrate metabolism to lipid biosynthesis and is highly relevant to cancer progression through its role in the altered MUFA metabolism. ACLY is a homotetrameric enzyme that catalyses the conversion of cytosolic citrate into acetyl‐CoA, which then serves as a central precursor for de novo fatty acid synthesis and cholesterol production via the mevalonate pathway [58, 59]. Increased production of acetyl‐CoA fuels the sequential activities of ACC, FASN, and SCD1, resulting in enhanced MUFA biosynthesis. Increased MUFA incorporation into membrane phospholipids promotes membrane biogenesis and fluidity and cancer cell proliferation while reducing PUFA abundance, thereby limiting lipid peroxidation and suppressing ferroptosis [60]. In addition to its metabolic function, ACLY‐derived acetyl‐CoA serves as a substrate for histone and protein acetylation, thereby regulating the expression of genes involved in lipid metabolism, cell cycle progression and oncogenic signalling [61, 62]. Because acetyl‐CoA availability directly influences lipid anabolism, changes in ACLY expression can greatly affect the membrane lipid composition, energy balance and proliferative capacity. Stable knockdown of ACLY has been shown to disrupt glucose‐dependent lipid synthesis, accompanied by an increase in membrane potential, reduced cytokine‐stimulated cell proliferation and resistance to cell death. These findings further emphasise the role of ACLY in supporting cancer cell survival [40]. ACLY has been found to be overexpressed in several cancers, including breast, lung, colorectal and prostate cancer. The ACLY gene showed significantly higher activity in lung adenocarcinoma samples than in lung tissue samples from patients with normal lungs [38]. ACLY is essential for lipid synthesis and is stabilised in cancer cells by acetylation at specific lysines, enhancing fatty acid production and tumour growth [42]. This modification was increased in lung cancer and linked to lipid‐driven cell proliferation [42]. Collectively, these findings indicate that ACLY promotes metabolic reprogramming in cancer by sustaining lipid biosynthesis and supporting the high anabolic demands of rapidly proliferating tumour cells.

#### 4.2.2. ACC

ACC is an enzyme that relies on biotin as a cofactor and facilitates the carboxylation of acetyl‐CoA to synthesise malonyl‐CoA. ACC has two isoforms: ACC1 and ACC2. ACC1 is primarily expressed in lipogenic tissues, where it facilitates the committed step in long‐chain fatty acid biosynthesis. ACC2 is predominantly expressed in skeletal muscle and heart tissues [63]. ACC is overexpressed in numerous cancers. A study in which mammary epithelial‐specific ACC1 was deleted in breast cancer in vivo showed impaired tumour progression due to decreased cancer cell proliferation; at the same time, the authors also showed that knockout of ACC1 in breast cancer cell lines led to corresponding outcomes [64]. ACC inhibition has been shown to reduce FA synthesis and suppress tumour growth. A decreased proliferation rate was observed in two glioblastoma cell lines, U87 and U87 EGFRvIII, with dual ACC1/ACC2 inhibitor treatment and dual siRNA knockdown [43].

#### 4.2.3. Fatty Acid Synthase (FASN)

FASN is a multi‐enzyme complex monomer that comprises six distinct enzymatic grooves that produce SFA from acetyl‐CoA and malonyl‐CoA and NADPH as a cofactor [65]. Formerly known as OA‐519, FASN in human breast cancer cells was considered to be oncogenic [66]. FASN is found to be overexpressed in many cancers, mainly in breast and lung cancers. Overexpression of FASN was found to increase HER2 signalling; thus, inhibiting FASN with the small‐molecule tyrosine kinase inhibitor Lapatinib blocks phosphorylation of HER2 and EGFR in breast cancer cells, inducing apoptosis [67]. A study shows that silencing of FASN leads to enhanced activation of SREBP2 and increased expression of HMGCR in human hepatocellular carcinoma (HCC) cell lines [45]. Another study showed that FASN knockdown using FASN shRNA or the inhibitors C75 and Cerulenin reduced lymph node metastasis (LNM) in cervical cancer (CC), suggesting that FASN inhibition significantly reduces the migration of CC cells [68].

#### 4.2.4. SCD

The rate‐determining step in the biosynthesis of MUFA occurs in the ER. SCD functions by incorporating a cis–double bond between carbons 9 and 10 at the Δ position relative to the carboxylic group, using NADH as a reducing equivalent for desaturation [69]. To date, five isoforms of SCD have been identified: four mouse SCD isoforms (1–4) and two human isoforms. In humans, the most abundantly expressed isoform in the lipogenic tissue is SCD1, whereas SCD5 is expressed in the brain and pancreas [70]. RNAi and SCD inhibition with A939572 decreased cell proliferation in bladder cancer and showed SCD as a differentially expressed gene. SCD is upregulated in many cancers, and its inhibition can reduce cancer cell proliferation. Inhibition of SCD1 by A939572, combined with enzalutamide, which inhibits castration‐resistant prostate cancer (CRPC), leads to significantly reduced tumour growth through changes in tumour‐specific lipid desaturation, suggesting that the equilibrium between MUFA and SFA is essential to maintain the aggressive prostate cancer phenotype [47, 71]. Moreover, SCD1 was identified as playing a crucial role in regulating tumour‐initiating cells and the development of resistance to sorafenib through its influence on ER stress‐mediated differentiation in liver cancer [72]. Inhibition of SCD1 activity with the small‐molecule inhibitor CVT‐11127 in lung cancer activated AMPK and decreased ACC activity, corresponding to a reduction in glucose‐mediated lipogenesis. Furthermore, compromised SCD1 activity led to the downregulation of SFA synthesis [73].

#### 4.2.5. ELOVLs

ELOVLs are a group of enzymes that elongate the carbon chains of fatty acids into longer chains containing 18 carbon atoms or more, forming very long‐chain fatty acids (VLCFAs) using NADPH as a cofactor in the ER. Seven isoforms of this enzyme are found, and each of the elongases does the elongation of a specific type of fatty acid, and the ones involved for MUFAs and SFAs are ELOVL1, ELOVL3, ELOVL6 and ELOVL7 [27]. ELOVL1 is the major elongase found to elongate chain lengths C‐2O and higher, while ELOVL3, 6 and 7 are involved in the elongation of C16–C22 of MUFA as well as SFA [74]. TFs such as LXR, SREBP‐1 and PPARα regulate the expression of elongases [75]. Poor patient prognosis is observed for ELOVL1 and ELOVL3 expression in liver cancer. In the same study, ELOVL1 was found to interfere with immune checkpoint markers, such as PD‐1 and CTLA4, thereby affecting immunotherapy. The authors also found that ELOVL1 was associated with TP53 oncogenic mutation [50]. Increased ELOVL3 expression is associated with poor prognosis in liver cancer and can be an oncogene for HCC pathogenesis [51]. Similarly, ELOVL6 was also found to be upregulated in liver cancer, showing poor prognosis in patients, and its knockdown in HCC showed Akt activation and reduced cell proliferation, indicating the oncogenic activity of ELOVL6 [52]. ELOVL7 is upregulated in prostate cancer cells as it affects the de novo synthesis of androgens, and its knockdown reduced tumour growth [53]. Thus, ELOVL enzymes play a distinct role in various cancers and could be further implicated as prognostic markers.

#### 4.2.6. Long‐Chain Acyl‐CoA Synthetase (ACSL3)

ACSL3 is one of the enzymes found in the ER among the five ACSL families, including ACSL1 and ACSL(3–6). ACSL3, independent of lipid droplet formation, activates exogenous MUFAs such as OA and PA into fatty acyl‐CoA esters and incorporates them into membranes, displacing PUFAs and thereby helping protect cells against lipid peroxidation [76]. The ACSL3 enzyme is also linked to lipid droplet formation and is involved in the β‐oxidation of fatty acids for energy [77]. ACSL3 is overexpressed in several cancers, including lung, breast, pancreatic, prostate and colorectal cancers [78]. SREBP1, CREBP, LXR, PPARδ and OCT1 transcriptionally activate the ACSL3 gene, which is associated with androgen receptor (AR) signalling. OCT1, a TF that regulates ACSL3 and androgen signalling genes, acts as a predictive biomarker in prostate cancer and has been shown to reduce its in vivo growth [54].

### 4.3. MUFA and Cancer Hallmarks

MUFAs are increasingly recognised as important regulators of cancer biology, influencing several well‐established cancer hallmarks. The way cancer cells produce, use and respond to MUFAs influences these characteristics, including resistance to cell death, altered metabolism, invasion and metastasis, persistent proliferative signals and evasion of immune destruction (Table 3).

**Table 3:** Role of MUFA in carcinogenesis and different hallmarks of cancer.

Hallmark of cancer	Types of cancer	References
Sustaining proliferative signalling	Overexpression of CD36 in HCC	[79]
	CD36 activated ER‐ targeted genes through ERK1/2 (p‐ERK1/2) signalling in breast cancer cells	[80]
	RNAi of ACYL gene affected cell proliferation in lung cancer cells	[38]
	ACLY acetylation was observed in lung cancer cells inducing lipid‐driven cell proliferation	[42]
	Dual siRNA knockdown in glioblastoma cancer cells showed decreased cellular proliferation	[43]
	RNAi and SCD inhibition with A939572 showed decreased cell proliferation in bladder cancer	[47, 71]
	Blocking SCD1 with CVT‐11127 in lung cancer activated AMPK and reduced cell proliferation	[73]
	Increased ELOVL6 expression showed poor prognosis of liver cancer by Akt activation and increased cell proliferation	[52]
	Increased ACSL3 regulated by OCT1 showed increased prostate cancer cell proliferation in vivo	[54]
	ChREBP siRNA knockdown resulted in the inhibition of cell proliferation and apoptosis in colon cancer cell lines	[55]
	Overexpression of HER2 in breast cancer cells that results in oncogenic transformation by mTORC1 and PPARγ oncogenic signalling activity	[81]
	Oleic was found to stimulate proliferation, secretion of MMP‐9 in breast cancer cells	[82]
Activating invasion and metastasis activating invasion and metastasis	Overexpression of CD36 in CRC	[83]
	Knockdown of SLC27A4 decreased invasion and migration in breast cancer subtypes	[84]
	FASN knockdown deceased lymph node metastasis in cervical cancer	[68]
	SCD1 overexpression led to sorafenib resistance in HCC leading to cancer stemness	[72]
	Activation of PPARδ plays a role in driving liver metastasis and promoting the expansion of colonic cancer stem cells (CSCs) in response to a high‐fat diet	[56]
	ChREBP induced GLUT1 overexpression was linked poor overall survival and invasiveness in liver cancer	[55]
	Inhibiting SCD1 expression inhibits NF‐κB pathway thereby eliminating CSCs, and blocks tumour initiation in ovarian CSCs	[85]
	SREBP1 promote EMT and metastasis in oesophageal squamous cell carcinoma via Wnt/β catenin signalling pathway	[86]
	ACOT12 was found downregulated in HCC, enhancing metastasis and poor prognosis	[87]
	Elevated levels of ACLY induces EMT and metastasis in lung cells on exposure to PM2.5	[41]
	In lung cancer cells EGFR signalling phosphorylates SCD1 on Y55 increases MUFA levels mediating metastasis	[88]
	ACC1 drives metastasis in breast cancer by activating Smad2	[44]
	FASN activates ErbB1, 2 and 4 during EMT in breast cancers encouraging metastasis	[89]
	SCD1 silencing reduces ERK1/2 signalling, inhibits β‐catenin target gene activation effecting metastasis in breast cancer cells	[49]
	ZEB1 is found to regulate EMT and MUFA:PUFA composition genes in breast cancer cells	[90]
	MBOAT1 and MBOAT2 are enzymes that are found to be suppressors of ferroptosis and phospholipid remodelling independent of GPX4 or FSP1	[91]
	Inhibiting FASN with osthole which also interferes with the HGF/c‐Met‐mediated EMT inhibits breast cancer metastasis	[92]
Resisting cell death (apoptosis evasion)	Overexpression of FASN was found to increase HER2 signalling thereby protecting cells against apoptosis.	[67]
	Inhibition of FASN by orlistat decreased proliferation of human pancreatic cancer cells and induced apoptosis.	[93]
	SCD1 inhibitor A939 shwed induced apoptosis in colorectal cancer cells in vivo	[94]
	Poor patient prognosis is observed for ELOVL1 and ELOVL3 expression in liver cancer resisting immunotherapy	[50, 51]
	SREBP‐1 c modulated decreased MUFA genes expression when treated with MAP kinase inhibitor, PI 3‐kinase inhibitor, wortmannin diminished transformation capacity in breast cancer cells	[95]
	SCD1 and HIF‐2 α (TF) were found upregulated in clear cell renal cell carcinoma (ccRCC), KD of either of them led cells prone to apoptosis	[57]
	Sorafenib activate AMPK and mTOR signalling to inhibit SREBP1c thereby SCD and sensitise cells to ferroptosis in liver cancer	[96]
	SLC25A22 downregulation sensitises pancreatic ductal adenocarcinoma (PDAC) cells to ferroptosis by AMPK signalling	[97]
	Silencing USP2a which is highly expressed with enhanced activity of fatty acid metabolism and down‐regulation of a cell death gene can induce apoptosis in prostate cancer cells	[46]
	Downregulating SCD1 and NR4A1 gene expression by FBW7 through c‐Myc/TXNIP axis induces ferroptosis in pancreatic cancer cells	[98]
	Cucurbitacin B (CuB) inhibits ACLY gene expression and induces apoptosis in prostate cancer cells	[99]
Inducing angiogenesis	SREBP1 overexpression in the colon cancer cell line HT29 enhances angiogenesis, increases ROS and promotes MMP7 expression	[10]
Evading growth suppressors	Overexpression of SCD1 activity through ChREBP increases MUFA facilitating metastasis of CRC cells by repressing PTEN	[48]

## 5. Role of MUFA in Ferroptosis

Ferroptosis is a form of controlled cell death characterised by excessive accumulation of lipid peroxide to potentially toxic levels and is mechanistically different from both apoptosis and necrosis [100, 101] (Table 4). This type of cell death is characterised by the oxidative degradation of polyunsaturated phospholipids within cellular membranes, a process tightly regulated by intracellular iron availability and antioxidant defence systems, particularly glutathione and glutathione peroxidase 4 (GPX4). Unchecked lipid peroxidation compromises membrane integrity, leading to cell death. Ferroptosis plays vital roles in a number of biological contexts, including ischaemia‐reperfusion injury, neurodegeneration and cancer. Due to its unique metabolic and redox dependences, ferroptosis is increasingly being recognised as a potential therapeutic target [101, 104–106]. MUFAs have superior chemical stability and are more resistant to peroxidation than PUFAs because they have a single double bond. When incorporated into membranes, MUFAs restrict the pool of substrates available for lipid peroxidation, thereby reducing the cell vulnerability to ferroptosis. MUFA‐enriched membranes exhibit reduced accumulation of lipid peroxides and increased resistance to ferroptosis, demonstrating this protective effect in both *in vitro* and *in vivo* settings [9, 107]. Ferroptosis is suppressed by the phospholipid‐modifying enzymes MBOAT1 and MBOAT2. They inhibit ferroptosis by phospholipid remodelling independent of GPX4 or FSP1. These proteins have been found to be transcriptionally upregulated by sex hormone receptors, including the AR and the oestrogen receptor (ER). Co‐treatment with hormone receptor antagonists (ER or AR inhibitors) and ferroptosis‐inducing agents leads to a significant reduction in tumour growth in ER‐positive breast cancer and AR‐positive prostate cancer models, even in cases where the tumours display resistance to standard hormone therapies [91]. As previously described, SCD1 regulates the synthesis of MUFA in cells by converting SFAs into their monounsaturated forms. A balanced supply of MUFAs, whether produced internally or obtained from outside sources, is crucial for maintaining the structural integrity of cellular membranes and for shielding cells against ferroptotic cell death. Inhibition of SCD1 increases sensitivity to ferroptotic cell death, highlighting the enzyme’s crucial role in MUFA‐mediated defence [108, 109]. ACSL3 activate FFAs to be incorporated by the membrane‐bound O‐acyltransferase domain containing 1/2 (MBOAT1/2) into phospholipids in the cell membrane. Inhibition of ACSL3 prevents the execution of ferroptosis [110, 111]. Upon inhibition of system Xc, cystine starvation or GPX4 inhibition, it was shown that the supplementation of cells with exogenous MUFAs leads cells into a state that makes them resistant to ferroptosis. Treatment with exogenous OA reduces the levels of H‐PUFAs in the membrane or stimulates a downstream signalling pathway to exert an indirect antioxidant effect [112]. The downregulation of the mitochondrial glutamate transporter SLC25A22 makes pancreatic ductal adenocarcinoma (PDAC) cells more susceptible to ferroptosis. SLC25A22 expression contributes to resistance against ferroptosis by increasing GSH levels via NADPH synthesis and by increasing anti‐ferroptotic MUFAs through AMPK‐driven SCD expression [97]. The protective effect of MUFA against ferroptosis has also been discussed by Tesfay et al. [113] and Yi et al. [114], whose studies highlight the significance of combination therapy using ferroptosis inducers with SCD1 inhibitors in cancer therapy. In conclusion, MUFAs play a crucial role in regulating ferroptosis resistance by altering the structure and biochemistry of lipid membranes.

**Table 4:** Comparative features of apoptosis, necrosis and ferroptosis, highlighting their principal morphological and biochemical characteristics.

Feature	Apoptosis	Necrosis	Ferroptosis	References
Nature of cell death	Programmed and regulated	Typically, non‐programmed, uncontrolled	Regulated, non‐apoptotic	[102, 103]
Main trigger	Developmental cues, DNA damage and stress	Severe injury, toxins, infection and extreme stress	Iron‐dependent lipid peroxidation, failure of antioxidant defence	
Energy requirement	ATP‐dependent	Often associated with ATP depletion	Not classically ATP‐dependent; driven by redox imbalance	
Cell morphology	Cell shrinkage, membrane blebbing, apoptotic bodies	Cell swelling, membrane rupture, lysis	Small/shrunken mitochondria, increased membrane density, reduced/absent cristae	
Nuclear changes	Chromatin condensation, DNA fragmentation	Nuclear dissolution/disintegration	No classic apoptotic DNA fragmentation pattern	
Plasma membrane	Preserved until late stages	Lost early	Ultimately compromised after lipid damage; not an early blebbing process	
Inflammation	Minimal or absent	Prominent	Can promote inflammatory signalling via lipid peroxidation products nature	
Key biochemical hallmark	Caspase activation	Cell swelling and release of intracellular contents	Lipid peroxidation and iron accumulation	

## 6. Targeting MUFA Metabolism in Cancer

The metabolism of MUFAs is intricately linked to various metabolic pathways, including the oxidative stress response and ferroptosis, positioning MUFAs as targets for cancer. Current research is increasingly focused on harnessing the MUFA metabolism to exploit the metabolic vulnerabilities observed in cancer cells. This approach includes strategies such as inhibiting key enzymes like SCD1 and FASN, modifying lipid composition to induce oxidative stress, and exploiting cancer cells’ reliance on MUFAs for survival. These strategies represent potential avenues for developing innovative anti‐cancer therapies by disrupting the altered lipid metabolic landscape present in tumours.

Cerulenin‐mediated inhibition of FASN blocked fatty acid synthesis and suppressed tumour growth, an effect that was partially reversed by palmitate supplementation. These findings demonstrate that FASN plays a critical role in driving breast cancer progression, highlighting its potential as a novel therapeutic target [66]. Osthole, a naturally occurring coumarin derivative, is a potent inhibitor of FASN activity. In breast cancer cells, osthole inhibits HGF/c‐Met‐mediated epithelial–mesenchymal transition (EMT) and cell migration and increases apoptosis. Furthermore, it prevents c‐Met phosphorylation and expression in breast cancer cells, highlighting its potential to inhibit breast cancer metastases [92]. Combining ferroptosis‐inducing agents and apoptosis‐activating agents with chemotherapy can also be used as a therapeutic. Studies have demonstrated that FBW7 exerts anticancer effects by promoting ferroptosis and apoptosis. This occurs through the downregulation of SCD1 and NR4A1 and suppression of lipid peroxidation and glucose metabolism via the c‐Myc/TXNIP axis, ultimately enhancing cytotoxicity in pancreatic cancer [98]. A natural compound, cucurbitacin B (CuB), an ACLY inhibitor, selectively inhibits cell growth in prostate cancer while causing apoptosis without affecting normal cells. Implementing that CuB represents a potential chemopreventive agent for the treatment of prostate cancer [99]. All of the previous findings point to MUFAs being active participants in altering the physiology of cancer cells to withstand treatment rather than passive metabolic byproducts. Modifying MUFA metabolism, either by blocking MUFA absorption pathways or by targeting SCD1, is a viable strategy to enhance treatment efficacy and re‐sensitise cancer cells to traditional chemotherapy. In summary, MUFA metabolism plays a pivotal role in driving chemoresistance and offers novel therapeutic targets in cancer. Targeting MUFA biosynthesis and function can effectively disrupt the metabolic adaptations of tumours, offering a promising avenue for enhancing therapeutic efficacy (Figure 3) (Table 5).

**Figure 3:**
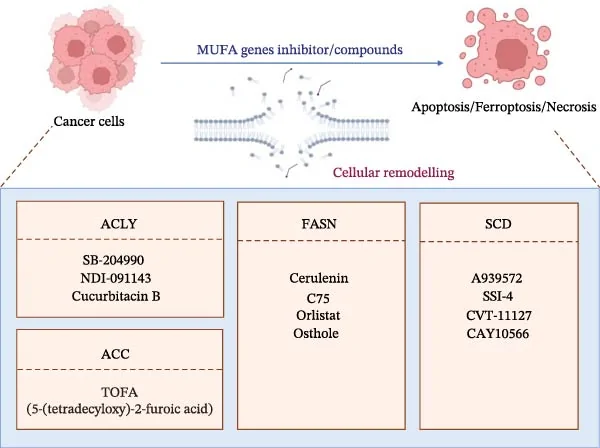
Therapeutic implication of MUFA in cancer. Targeting MUFA biosynthesis and function in cancer cells by inhibiting different MUFA genes can effectively disrupt the metabolic adaptations of tumours, thereby enhancing therapeutic efficacy.

**Table 5:** Therapeutic potential of targeting MUFA metabolism in cancer.

Target gene	Inhibitor/compound	Mechanism of action	Type of cancer	References
ACLY	SB‐204990 and NDI‐091143	Dose‐dependent (and time‐dependent) suppression of two‐dimensional and three‐dimensional cell growth, induces apoptosis and reduces colony formation and cell invasion	Thyroid cancer	[115]
	Cucurbitacin B	Inhibits ACLY phosphorylation and acts as a chemopreventive agent	Prostate cancer	[99]
ACC	TOFA	Prevented GPX4 loss and suppressed FIN56 lethality	Oncogenic RAS expressing osteosarcoma, lung adenocarcinoma, fibrosarcoma and engineered BJ cell lines.	[116]
FASN	Cerulenin	Inhibits acylglycerol and thereby lipid synthesis	Breast cancer	[66]
	Lapatinib	Blocks phosphorylation of HER2 and epidermal growth factor receptor inducing apoptosis	Breast cancer	[67]
	C75 and cerulenin	Knockdown of FASN using FASN shRNA or inhibition by inhibitors significantly reduced lymph node metastasis (LNM) in vivo	Cervical cancer	[68]
	Orlistat	Decreased proliferation and induced apoptosis	Pancreatic cancer	[93]
	MAP kinase inhibitor, PI 3‐kinase inhibitor and wortmannin	SREBP‐1 c modulation of FASN showed decreased transformation capacity	Breast cancer	[92]
	Cerulenin	Inhibition of FASN inhibits expression of ErbB1−4, receptors which promotes EMT	Breast cancer cell line MCF‐7‐MEK5	[89]
	Osthole	Inhibition of FASN inhibited c‐Met phosphorylation acting as a potential target of HGF/c‐Met‐induced EMT progression	Breast cancer	[92]
SCD	A939572 combined with enzalutamide	Reduced tumour growth	Prostate cancer	[71]
	SSI‐4 combined with sorafenib	SCD1 suppression results in enhanced sensitivity to sorafenib	Liver cancer	[72]
	CVT‐11127	Leads to the activation of AMP‐activated kinase (AMPK) and decreased ACC thereby decreased glucose mediated lipogenesis	Lung cancer	[73]
	A939	Delayed tumour growth and further inhibits ceramide biosynthesis in vivo	Colorectal cancer	[94]
	CAY10566	Inhibition of SCD1 inhibits NF‐κB pathway blocking tumour initiation	Ovarian CSCs	[85]
	Sorafenib	Activate AMPK which coordinates with mTOR and inhibits SREBP1c indirectly inhibiting SCD1	Liver cancer	[96]
	FBW7 (FBXW7)	Downregulates SCD1 and NR4A1 gene expression by inhibiting lipid peroxidation via c‐Myc/TXNIP axis and enhancing the cytotoxicity	Pancreatic cancer	[98]
mTORC1 and PPARγ	Docosahexaenoic acid	Reduced oncogenic transformation	Breast cancer	[81]

## 7. Discussion

Alterations in lipid metabolism have emerged as a hallmark of cancer, contributing significantly to tumour initiation and progression. These metabolic shifts are often driven by changes within the tumour microenvironment, which reprogramme lipid processing in cancer cells. SFAs, MUFAs and PUFAs are essential lipid components that play pivotal roles in maintaining membrane lipid homeostasis. The specific distribution of fatty acids within cellular membranes is critical for regulating key physiological processes, including membrane fluidity, structural integrity, signal transduction and protection against oxidative stress and reactive oxygen species. A balanced SFA:MUFA:PUFA ratio is important for protecting cells from lipotoxicity. In cancer cells, these ratios are significantly altered [3, 4, 7]. The complex role of MUFA in tumour biology has attracted considerable interest. This is especially true when considering ferroptosis, a regulated form of cell death caused by iron‐dependent lipid peroxidation. MUFAs have a single double bond, which gives them more oxidative stability than PUFAs, which are more susceptible to peroxidation because of their numerous double bonds. Their ability to shield cancer cells from ferroptotic stress is supported by this unique structural characteristic. MUFA metabolism serves a pivotal role in cancer growth by increasing their metastatic potential for proliferation and protecting them from anti‐tumour compounds [16].

The incorporation of a higher number of MUFAs into cancer cells occurs due to the upregulation of MUFA‐associated genes. These genes are regulated by various TFs and signalling cascades. In various cancers, genes involved in the MUFA production pathway, such as ACLY, ACC, FASN, SCD, ELOVLs and ACSL, are upregulated. Experimental studies using RNAi to knock down these genes showed decreased proliferation and metastasis in breast, lung, liver and colorectal cancers. Also, studies have shown that small‐molecule inhibitors such as cerulenin, sorafenib, and CuB inhibit gene expression directly or indirectly, thereby reducing cancer proliferation [117]. However, the precise mechanisms by which mutations drive activation of oncogenic pathways, disrupt gene expression, and alter the interplay between MUFA metabolism and ferroptotic pathways remain poorly understood and warrant further investigation. Targeting MUFA metabolism by SCD1 inhibition in cancer cells has been shown to re‐sensitise them to ferroptosis inducers, such as RSL3 and erastin. By disrupting MUFA synthesis, SCD1 inhibitors promote the accumulation of lipid peroxides and trigger cell death by ferroptosis. Numerous studies have shown that targeting these genes with inhibitors decreases cell proliferation and tumour growth. Genes such as SCD and FASN could be inhibited by A939572, and osthole could act as a therapeutic in treating various cancers. The efficacy of these inhibitors in targeting the genes should be prioritised while considering off‐target effects and minimising harm to healthy tissues [92, 118].

Despite substantial progress, several important questions remain unresolved. Most mechanistic studies have been performed in cultured cell lines or animal models, while clinical evidence validating MUFA‐targeted therapeutic strategies remains limited. Moreover, the contribution of dietary MUFAs compared with de novo‐synthesised MUFAs to tumour progression remains controversial, with epidemiological studies often reporting inconsistent findings due to differences in dietary habits, tumour heterogeneity and metabolic context. Similarly, the roles of individual lipogenic enzymes may differ between cancer types and disease stages, indicating that therapeutic strategies targeting MUFA metabolism will likely require careful patient stratification.

Another emerging area of interest is the interaction between lipid metabolism and the tumour microenvironment. Recent studies indicate that adipocytes, stromal cells and immune cells actively influence fatty acid availability within tumours, thereby shaping lipid metabolic reprogramming and therapeutic responses [2]. Understanding how these interactions regulate MUFA metabolism may provide new opportunities for combination therapies involving metabolic inhibitors, ferroptosis inducers, chemotherapy, immunotherapy or targeted agents.

MUFAs constitute a critical lipid line of defence in cancer, mediating resistance to ferroptosis through structural, enzymatic and transport‐dependent mechanisms. Therapeutic modulation of MUFA biosynthesis or membrane incorporation could serve as a novel strategy to amplify ferroptosis and optimise anti‐cancer treatment outcomes. Further research is needed to delineate the tissue‐specific regulation of MUFA metabolism and its implications for tumour progression, therapy resistance and immune modulation.

## 8. Conclusion

MUFA metabolism has emerged as a fundamental determinant of cancer progression, extending far beyond its conventional role in lipid storage and membrane synthesis. Increasing evidence demonstrates that enhanced MUFA biosynthesis supports metabolic reprogramming by promoting membrane phospholipid remodelling, maintaining redox homeostasis and reducing susceptibility to ferroptosis. These metabolic adaptations enable cancer cells to sustain rapid proliferation, survive oxidative stress and acquire invasive and metastatic characteristics. Central regulators of de novo lipogenesis, including ACLY, ACC, FASN, SCD1, ELOVLs and fatty acid transporters, function in concert with oncogenic signalling pathways to establish a lipid metabolic environment that favours tumour growth. Recent studies further indicate that pharmacological inhibition of MUFA biosynthetic enzymes restores sensitivity to ferroptosis and enhances the efficacy of existing anticancer therapies. Collectively, the available evidence highlights MUFA metabolism as a critical metabolic vulnerability in cancer and supports the development of therapeutic strategies that target lipid metabolic reprogramming to improve cancer treatment outcomes.

## 9. Future Perspectives and Limitations

Over the years, several studies have enriched our understanding of the role of MUFA metabolism in cancer progression. However, several important questions remain unanswered. The majority of the studies have extensively used in vitro cancer cell line models and preclinical animal studies, while clinical validation of MUFA‐targeted therapies remains limited. The molecular mechanisms linking MUFA metabolism with ferroptosis, tumour heterogeneity, immune regulation and therapeutic resistance also require further investigation across different cancer types. Additionally, the interplay between MUFA metabolism, mitochondrial function, oxidative stress and lipid remodelling remains to be elucidated. Therefore, future studies integrating lipidomics, metabolomics, single‐cell sequencing and spatial transcriptomics are expected to provide a more comprehensive understanding of these metabolic networks. In addition, the development of selective inhibitors targeting enzymes such as ACLY, FASN and SCD1, as well as lipid transporters, either alone or in combination with ferroptosis‐inducing agents, immunotherapy or conventional chemotherapy, warrants further evaluation in clinical settings. A better understanding of the context‐dependent regulation of MUFA metabolism may facilitate the identification of predictive biomarkers and enable the development of personalised therapeutic strategies. Despite current limitations, continued investigation into MUFA‐driven metabolic reprogramming is likely to provide new opportunities to improve cancer diagnosis, prognosis and treatment.

## Author Contributions

**Sneha D. Baadkar**: writing – original draft, formal analysis. **Amoolya Kandettu**: writing – review and editing, writing – original draft. **Swagat Ray**: writing – review and editing. **Sanjiban Chakrabarty**: writing – review and editing, writing – original draft, data curation, conceptualisation.

## Funding

This study was funded by the Science and Engineering Research Board (YSS/2015001051 and CRG/2022/004681), Board of Research in Nuclear Sciences (202203HLC19RP07105‐BRNS) and Indian Council of Medical Research (IIRPIG‐2023‐0000845). The authors gratefully acknowledge infrastructure funding from DST‐FIST, K‐FIST, TIFAC‐CORE, DBT‐BUILDER (BT/INF/22/SP43065/2021), VGST Karnataka, Manipal School of Life Sciences and Manipal Academy of Higher Education, Manipal, Karnataka.

## Conflicts of Interest

The authors declare no conflicts of interest.

## Data Availability

Data sharing is not applicable to this article as no datasets were generated or analysed during the current study.
